# Screening of diabetes mellitus among people living with HIV

**DOI:** 10.1002/jia2.25335

**Published:** 2019-06-28

**Authors:** Jan Brož

**Affiliations:** ^1^ Department of Internal Medicine Second Faculty of Medicine University Hospital Motol Charles University Prague Czech Republic

**Keywords:** diabetes mellitus, virologically suppressed PLHIV, antiretroviral therapy, comorbidities, Asia‐Pacific region

Dear Editor,

The article published by Han WM *et al*. 1 presented the results of a multicentric longitudinal study exploring the incidence of new‐onset diabetes mellitus (DM) among people living with HIV (PLHIV), after combination antiretroviral therapy (cART) initiation in several countries of the Asia and Pacific regions. Of the 1927 screened subjects there were 127 PLHIV (7%) who had DM after cART with an incidence rate of 1.08 per 100 person‐years under a median follow‐up time of 5.9 years. The authors noted that the DM incidence found among PLHIV in similar studies across the world varies from 4.7/100 PYS 2 to 0.44/100 PYS 3 and they emphasized the importance of monitoring and routine screening for non‐communicable diseases including DM among PLHIV.

The study team collected and analyzed a large amount of data, leading to an interesting and important debate. Nevertheless, I would like to make one comment that may contribute to a further and more detailed discussion of the issue.

A patient with DM in the study was defined, according to modified criteria for DM diagnosis of the American Diabetes Association, as having a single measurement showing fasting blood glucose (FBG) ≥126 mg/dL, HbA1C ≥6.5%, a two‐hour plasma glucose level after oral glucose tolerance test (OGTT) ≥200 mg/dL, or a RPG ≥200 mg/dL. Of the 127 PLHIV with DM in the study, 117 met the FBG criteria, 9 met the HbA1C criteria and 1 met the OGTT criteria for DM.

However, the sensitivity of the screening tests used in the study differs substantially and significantly favours HbA1c (≥6.5%) and OGTT to FBG. The study conducted in Vietnam, which focused on the comparison of different DM screening tests with a study population of 3523 individuals, showed the prevalence of DM based on the HbA1c test 9.7%, while DM prevalence based on the FBG test was only 6.3% 4. The Taiwanese study with 689 participants showed DM prevalence based on HbA1c, a two‐hour plasma glucose level after OGTT, and FBG of 17.9%, 19% and 3.8% 5.

There is a discrepancy between the sensitivity of the DM screening tests and the portions of patients diagnosed by each of them in the study by Han *et al*., with most of the DM patients diagnosed by FBG. This fact strongly suggests that not all patients underwent all of the test, with the test choice rather down to the discretion of the study investigators, thus a portion of the study subjects could be screened by a more sensitive test than the others. Such inconsistency could substantially and negatively influence the accuracy of the study results and may also contribute to an explanation of result variances of this 1 and the above‐mentioned studies 2, 3, as each of them used different DM definition.

With great respect, I suggest taking these comments into consideration if the continuation of this important study is planned.

## Competing interests

The author has no conflicts of interest to declare.
